# Evaluation of orange peel for biosurfactant production by *Bacillus licheniformis* and their ability to degrade naphthalene and crude oil

**DOI:** 10.1007/s13205-015-0362-x

**Published:** 2016-02-04

**Authors:** Arthala Praveen Kumar, Avilala Janardhan, Buddolla Viswanath, Kallubai Monika, Jin-Young Jung, Golla Narasimha

**Affiliations:** 1 Applied Microbiology Laboratory, Department of Virology, Sri Venkateswara University, Tirupati, India; 2 Department of Plant Sciences, Hyderabad Central University, Hyderabad, 500046 India; 3 Department of Environmental Engineering, College of Engineering, Yeungnam University, 280 Daehak-Ro, Gyeongsan, Gyeongbuk 712-749 South Korea; 4 Department of BioNanotechnology, Gachon University, San 65, Bokjeong-Dong, Sujeong-Gu, Seongnam-Si, Gyeonggi-Do 461-701 Republic of Korea

**Keywords:** *Bacillus licheniformis*, Orange peels, Emulsification activity, Lipopeptide, NMR analysis

## Abstract

A Gram-positive bacterium was isolated from mangrove soil and was identified as *Bacillus licheniformis* (KC710973). The potential of a mangrove microorganism to utilize different natural waste carbon substrates for biosurfactant production and biodegradation of hydrocarbons was evaluated. Among several substrates used in the present study, orange peel was found to be best substrate of biosurfactant yield with 1.796 g/L and emulsification activity of 75.17 % against diesel. Fourier transform infrared spectroscopy analysis of biosurfactant compound revealed that the isolated biosurfactant is in lipopeptide nature. The ^1^H-NMR of the extracted biosurfactant from *B. licheniformis* has a doublet signal at 0.8–0.9 ppm corresponding to six hydrogen atoms suggests the presence of a terminal isopropyl group. The spectra showed two main regions corresponding to resonance of α-carbon protons (3.5–5.5 ppm) and side-chain protons (0.25–3.0 ppm). All the data suggests that the fatty acid residue is from lipopeptide. From the biodegradation studies, it concluded that the biosurfactant produced by *B. licheniformis* further can add to its value as an ecofriendly and biodegradable product.

## Introduction

Environmental pollution with petroleum and petrochemical products has attracted much attention in recent decades. Microorganisms having the ability to degrade many poly aromatic hydrocarbons (PAHs) have been explained, and their mechanisms of action have been studied (Cerniglia 1992). The bioremediation of soils contaminated with PAHs is limited by the poor availability of these hydrophobic contaminants to microorganisms (Mihelcic et al. 1993). The presence of distinct types of automobiles and machinery has engendered in an increase in the use of lubricating oil. Spillage of used motor oils such as diesel or jet fuel contaminates our natural environment with hydrocarbon (Abioye et al. 2012). The illegal dumping of used motor oil is an environmental hazard with global ramifications (Blodgett 2001). Used motor oil contains metals and heavy polycyclic aromatic hydrocarbons (PAHs) that could contribute to chronic hazards including mutagenicity and carcinogenicity (Hagwell et al. 1992; Boonchan et al. 2000). Microbial surface active agents or biosurfactants are extracellular products consisting of hydrophobic and hydrophilic moieties and tend to interact with surfaces of different polarities and reduce the surface and interfacial tension of solutions, facilitating hydrocarbon uptake and emulsification/dispersion. They can improve the bioavailability of hydrocarbons to the microbial cells by increasing the area of contact at the aqueous–hydrocarbon interface. This increases the rate of hydrocarbon dissolution and their utilization by microorganisms (Singh et al. 2007; Satpute et al. 2010; Perfumo et al. 2006).

Generally biosurfactant production are using expensive carbon sources, it could be replaced with cheaply available natural raw materials. Agro-industrial wastes are with high contents of lipids and carbohydrates; it can be used as a carbon source for biosurfactant production (Makkar and Cameotra 2002). Mangroves ecosystems are the plant communities occurring in inter-tidal zones along the coasts of tropical and sub-tropical countries. Mangroves represent a rich and diverse living resource and are valuable to both the economy and protection of coastal environments. They have been considered as significant sinks for pollutions from freshwater discharges and from contaminated tidal water (Bernard et al. 1996; Reda and El Nagar 2009). However, the reports on biosurfactants produced by mangrove sediment microorganisms have been limited to date (Saimmai et al. 2012a, b, c). The objective of the present study was to produce biosurfactant from natural waste substrates by *Bacillus licheniformis* and evaluate their ability to degrade naphthalene and crude oil.

## Materials and methods

### Isolation and identification of biosurfactant producing microorganisms

100 g of mangrove sediment from coastal region of Kakinada, Andhra Pradesh, India was collected at a depth of 0–5 cm. Initially, the bacterial consortium was enriched by adding 1 g of soil sample to 50 ml of minimal salt medium (MSM) in a 250 ml Erlenmeyer flask containing 2 % glycerol as a carbon source (Arutchelvi and Doble 2010). Minimal salt medium composition (MSM) g/L: NaNO_3_—2.5, K_2_HPO_4_—1.0, KH_2_PO_4_—0.5, MgSO_4_—0.5, KCl—0.1, FeSO_4_—0.01, CaCl_2_—0.01, Glucose—30, pH—6.8 ± 0.2. The flasks were incubated at 150 rpm at 30 °C for 5 days. Thereafter, 1 ml aliquot of the culture broth was aliquoted to a fresh 50 ml MSM in a 250 ml flask and incubated under the same conditions as described above. This procedure was repeated three times. 1 ml of enriched cultures were diluted in a sterile 0.85 % saline solution and plated on nutrient agar plates for the isolation of microorganisms. Morphologically distinct colonies were re-isolated by transfer to nutrient agar plates thrice to obtain pure cultures and were subsequently gram-stained and selected for biochemical characterization and biosurfactant screening. Pure cultures were maintained in nutrient agar slants and stored at −20 °C.

### Molecular characterization of mangrove bacterial isolate

16S rRNA sequencing was performed through an external agency (Macrogen Korea, Korea). The resulting sequences were compared with sequences in the GenBank database of NCBI using the BLAST network service (Altschul et al. 1997). Multiple sequence alignments were carried out using ClustalW and a consensus neighbor-joining tree was constructed using Molecular Evolutionary Genetics Analysis (MEGA) software (Tamura et al. 2007).

### Screening of potent biosurfactant producer

The potential biosurfactant producer was screened by different methods hemolytic assay, drop collapsing test, oil displacement test, and lipase activity (Abu-Ruwaida et al. 1991; Youssef et al. 2004; Kiran et al. 2009).

### Selection of optimum natural waste substrates for biosurfactant production

To evaluate optimum natural waste substrates as carbon sources for biosurfactant production such as Citrus lambiri peels, Citrus medica peels, orange peels, banana peels and potato peels were in the place of glycerol in MSM at the same concentration of 2 %. All these waste substrates were dried and powdered before use. All the experiments were carried out in 250-ml Erlenmeyer flasks containing 50 ml of the medium inoculated with 2 % (v/v) inoculum and incubated at 30 °C for 120 h. Fermented broth was centrifuged at 10,000 rpm at 4 °C for 10 min for separation of supernatant.

### Biosurfactant production by using orange peel as carbon source

The effects of incubation time, temperature and substrate concentration were optimized. For all these experiments, 50 ml of the medium in 250-ml flask was inoculated with 2 % (v/v) of inoculum at 30 °C. The effect of incubation time on biosurfactant production was studied by incubating the medium between 1 and 5 days and the orange peel concentration was 2 %. The biosurfactant yield was evaluated at regular intervals of 24 h and the minimum period for maximum biosurfactant production was selected as the optimum incubation time. The biosurfactant production was also checked at different temperature conditions i.e., 25, 30, 35, 40 °C. The effect of concentration of orange peel was optimized between 1 and 5 %.

### Extraction of biosurfactant

Cell pellet was removed from the fermented broth by centrifugation at 10,000 rpm for 10 min. To precipitate the lipids and proteins, 6 N HCl was added to the culture supernatant to bring final pH of 2.0 and kept for overnight at 4 °C. Then the precipitated biosurfactants was extracted by adding equal volumes of ethyl acetate in a separating funnel and shaken vigorously. The organic phase was subjected to rota-evaporation for extraction of biosurfactant. The extracted biosurfactant was lyophilized and weighed for determination of biosurfactant yield.

### Study of growth and biosurfactant production

The analysis of growth and biosurfactant production were studied in different carbon substrates containing MSM in 250 ml conical flask with 50 ml working volume. Fermented broth were collected every alternate hour and checked for biomass, biosurfactant concentration and emulsification activity. Biomass was determined by dry cell weight method (Suwansukho et al. 2008). Emulsification activity was measured by adding equal volumes of hydrocarbon and culture supernatant mixed and vortexed for 2 min followed by incubation at room temperature for 24 h (Tabatabaee et al. 2005).

### Chemical characterization of biosurfactant

#### TLC analysis for crude biosurfactant

The extracted crude biosurfactants of *B. licheniformis* were examined using thin layer chromatography (TLC). Approximately 100 µL of each sample was applied to a preparative TLC (20 cm × 20 cm), with silica-gel 60 in chloroform–methanol–acetic acid (65:15:2, v/v/v). The lateral edges of the plate were sprayed with orcinol–H_2_SO_4_ (Monteiro et al. 2007) and developed at 100 °C for 5 min. Positive spots were scraped and then extracted with 3 ml of chloroform–methanol (2:1, v/v, twice), with centrifugation at 3000×*g* to remove the silica gel.

### High pressure liquid chromatography (HPLC) analysis

The purity of the separated component was tested by gradient elution high-performance liquid chromatography using a Waters C 18 column (4.6 × 250 mm) with a Waters 717 plus auto sampler and 2487 refractive index detector. The flow rate was 0.4 ml min^−1^, and the mobile phase used was acetone/acetonitrile (30:70 v/v). The injected sample volume was 20 µl.

### Identification of fatty acid and amino acid

The presence of fatty acid and amino acid in biosurfactant were identified by the method (Feignier et al. 1995). Protein content was determined (Lowry et al. 1951). Bovine serum albumin (BSA) was used as calibration standard.

### Fourier transform infrared spectroscopy (FTIR)

Fourier transform infrared spectroscopy (FTIR) was used for the molecular characterization of the biosurfactant. Lyophilized samples were analyzed by KBr pellet method.

### Biodegradation of PAH compounds (naphthalene) and crude oil

Bacterium was grown in a batch culture in a two different 250 ml flask containing 50 ml of MSM supplemented with PAH, i.e., Naphthalene (100 mg) in one flask and in another flask with crude oil (500 µl) as a sole carbon source and these flasks were inoculated with 2 % overnight broth culture. The conical flask was kept in shaker at 150 rpm at 37 °C for 10 days. At the regular time intervals, flasks were taken out and 1 ml culture was withdrawn for OD measurements at 600 nm and the same broth was used to estimate the end product of naphthalene biodegradation, i.e., salicylic acid and another flask containing broth for crude oil degradation studies.

### Analysis of naphthalene intermediate

The concentration of hydroxylated aromatic metabolites from naphthalene degradation was determined (Box 1983). As it was expected that the major metabolite in naphthalene degradation is salicylic acid, a standard graph was prepared by using sodium salicylate. The concentration of hydroxylated metabolic intermediates was estimated as salicylic acid equivalents in mg/ml.

### Estimation of crude oil degradation

Crude oil concentrations were determined as follows: crude oil samples were mixed with equal volume of petroleum ether to extract crude oil. Then the extracted crude oil was detected spectrophotometrically at 228 nm (Rahman et al. 2002).

### Bacterial adhesion to hydrocarbons (BATH assay)

The Cell hydrophobicity of the *B. licheniformis* with crude oil was measured using the BATH assay (Rosenberg et al. 1980). Hydrophobicity is expressed as the percentage of cell adherence to crude oil was calculated as follows:

$${\text{Percentage of bacterial adherence }}\left( \% \right) \, = \, \left( { 1- \left( {{{{\text{OD}}_{\text{aqueous\, phase}} } \mathord{\left/ {\vphantom {{{\text{OD}}_{\text{aqueous\,\, phase}} } {{\text{OD}}_{\text{original}} }}} \right. \kern-0pt} {{\text{OD}}_{\text{original}} }}} \right)} \right) \, \times { 1}00$$

### Statistical analysis

All the biosurfactant production experiments were carried out in three parallel replicates and the results presented were the average values of three determinations. The standard deviations for all measurements were less than 5 %.

## Results and discussion

### Isolation, screening and identification of biosurfactant-producing microorganism

**Fig. 1:**
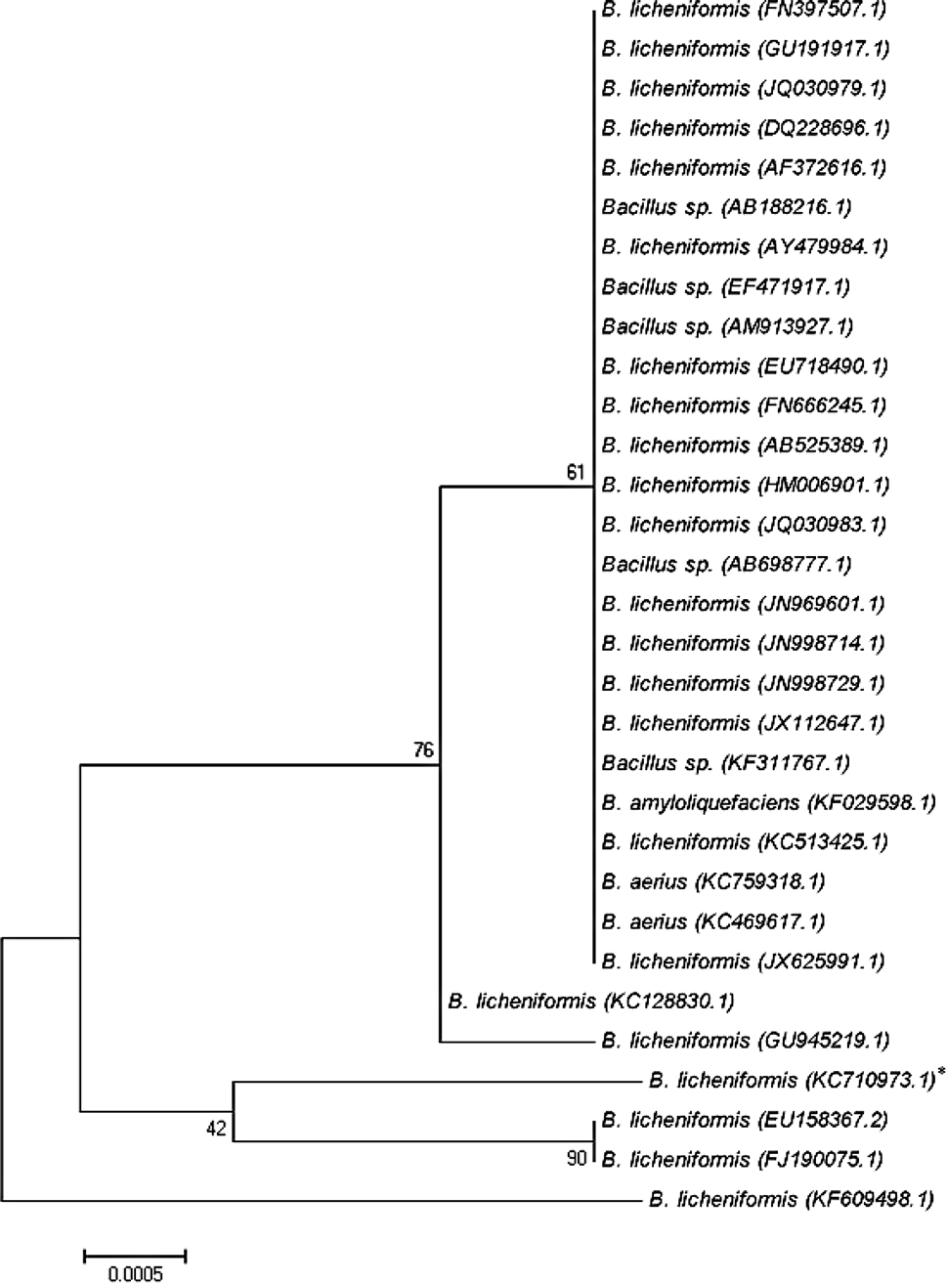
Phylogenetic tree of the strain *Bacillus licheniformis* and closest NCBI (BLASTn) strains based on the 16S rRNA gene sequences (neighbor joining tree method). The *scale bar* indicates 0.0005 nucleotide substitutions per nucleotide position. The *numbers* at node show the bootstrap values

A Gram-positive, endospore-forming, rodshaped bacterium with bacillar morphology was isolated from mangrove sediment of Kakinada mangrove region in Andhra Pradesh, India. The sequence of the 16S rRNA of the isolated bacteria demonstrated a high degree of similarity (99 %) with *B. licheniformis* (Fig. 1). The 16S rRNA sequences of strain *B. licheniformis* determined and submitted to GenBank database with accession numbers KC710973.

In the present study, the bacterial isolates were first screened with preliminary screening method for determining hemolytic activity. *B. licheniformis* showed good hemolytic activity. Blood agar lysis has been used to quantify surfactin (Moran et al. 2002; Rosenberg et al. 1980) and rhamnolipids (Johnson and Boese-Marrazzo 1980) and has been used to screen biosurfactant production by new isolates (Carrillo et al. 1996; Banat 1993). Carrillo et al. (1996) and Saimmai et al. (2012b) found an association between hemolytic activity and surfactant production, and they recommended the use of blood agar lysis as a primary method to screen biosurfactant production. None of the studies reported in the literature mention the possibility of biosurfactant production without a hemolytic activity (Moran et al. 2002; Johnson and Boese-Marrazzo 1980; Carrillo et al. 1996; Banat 1993). However, in some cases hemolytic assay excluded many good biosurfactant producers (Youssef et al. 2004).

The supernatant from *B. licheniformis* have shown good activity of 8 mm in diameter oil displacement activity and positive result in oil collapse method. The presence of biosurfactant was determined by oil displacement and clearing zone formation. The diameter of this clearing zone on the oil surface correlates to surfactant activity and hence satisfies oil displacement activity. Youssef et al. (2004), demonstrated that the oil spreading technique was very reliable method to detect biosurfactant production by diversified microorganisms.

**Fig. 2:**
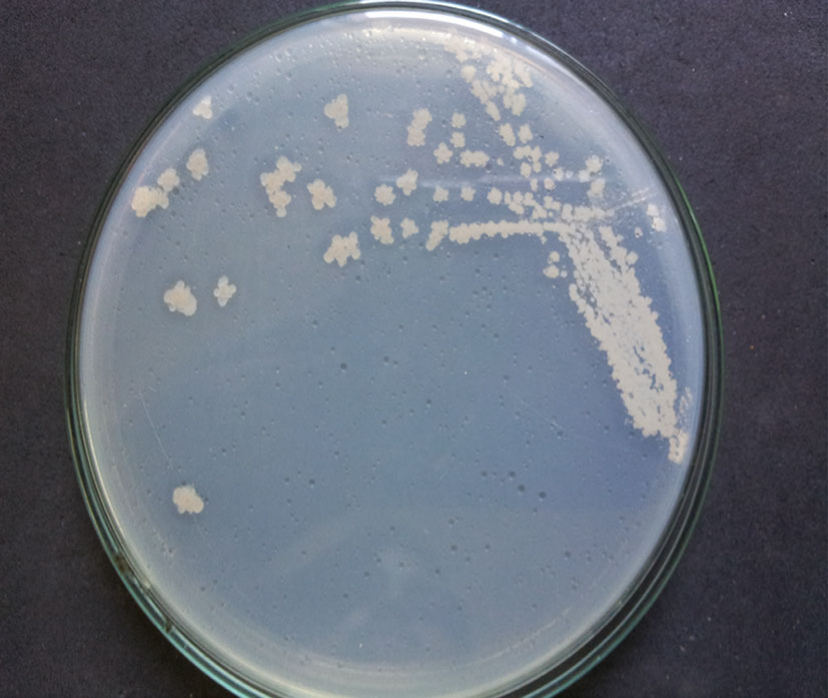
Lipolytic activity of mangrove bacterial isolate *B. licheniformis*

In the lipase screening activity, *B. licheniformis* showed positive lipolytic activity (Fig. 2). Inoculation of the bacterial isolates on Tributyrin agar plate produced a clear zone which indicates production of the enzyme, i.e., lipase. These results confirmed that the bacterial isolates were potential producers of surface active molecules. According to Kokare et al. (2007), lipase acts on water–oil surfaces and therefore it was suggested that actinomycetes showed the presence of lipases and are able to produce bioemulsifiers.

### Evaluation of biosurfactant production by *B. licheniformis*

Biosurfactant production from *B. licheniformis* with different natural waste substrates as carbon sources was studied using a mineral salt medium. The composition (g/L) of mineral salt medium prepared in distilled water was as follows: NH_4_NO_3_ (1.0); KH_2_PO_4_ (1.0); K_2_HPO_4_ (1.0); MgSO_4_·7H_2_O (0.2); CaCl_2_·2H_2_O (0.2) and FeCl_3_·6H_2_O (0.05). Among the five substrates tested, the highest percentage of emulsification and biosurfactant yield was obtained on using orange peels used as carbon source and the lowest emulsification index was found on Citrus lambiri peels (Table 1). Especially for Citrus lambiri and Citrus medica peels, the biosurfactant concentration of 0.876 and 0.809 g/L was recorded, respectively, but the emulsification activity of 22.56 and 26.04 % was measured, respectively. The yield was quite good using citrus peels but the emulsification activity was decreased. Similarly George and Jayachandran (2009), was reported when lime peelings used as a carbon source that the emulsification activity was not proportional to the biosurfactant yield. Based on these studies the orange peel was used for further studies as a carbon source to enhance the biosurfactant production.

**Table 1:** Biosurfactant produced from *B. licheniformis* with different natural waste substrates

Substrates	Biosurfactant concentration (g/L)*	Emulsification activity (%)*
Citrus lambiri peels	0.876 ± 0.068	22.56 ± 2.881
Citrus medica peels	0.809 ± 0.065	26.04 ± 2.713
Orange peels	1.295 ± 0.007	70.78667 ± 1.066
Banana peels	1.116 ± 0.103	66.49333 ± 0.854
Potato peels	1.058 ± 0.150	65.46667 ± 1.285

* Mean ± SD, no = 3

**Fig. 3:**
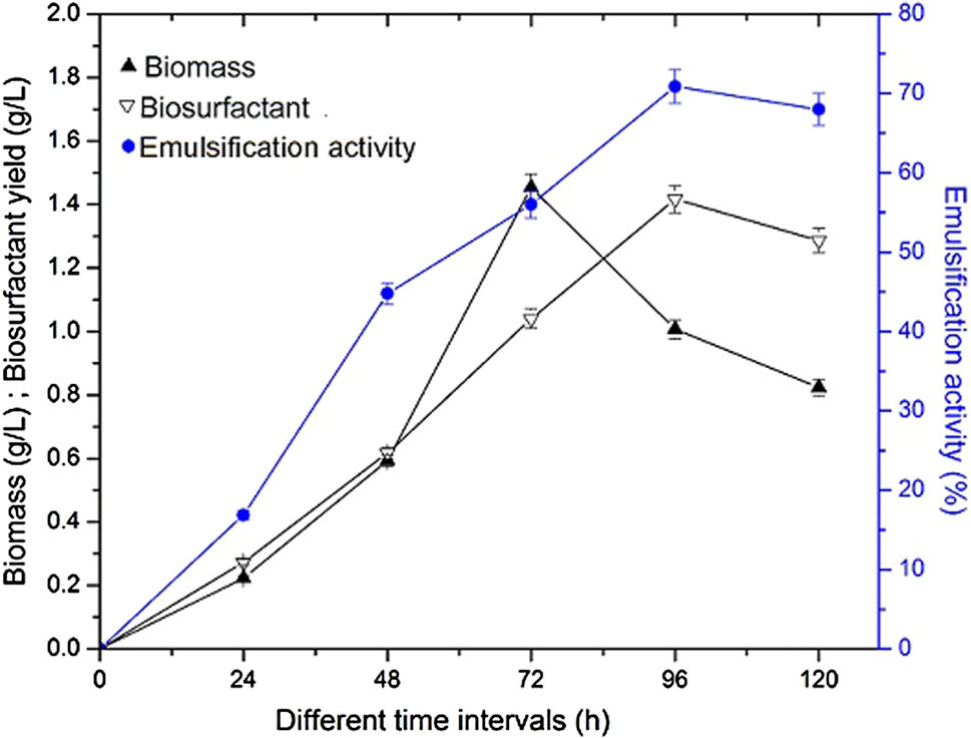
Effect of incubation time on biomass, biosurfactant yield and emulsification activity

The growth and biosurfactant production by *B. licheniformis* was monitored in mineral salts medium at different time intervals. *B. licheniformis* actively produced biosurfactants in the exponential growth phase and it was completed within 72 h there onwards decreased gradually in orange peel containing media (Fig. 3). Maximum emulsification activity and biosurfactant yield of 70.87 % and 1.41 g/L was obtained at 96 h of incubation, respectively (Fig. 3). The biosurfactant production was significantly increased with the increase in the orange peel concentration and obtained the maximum yield of 1.796 g/L and emulsification activity of 75.17 when 4 % orange peel as a carbon source (Table 2). The optimum temperature for maximum biosurfactant yield was found at 30 °C and there onwards the biosurfactant production was decreased with increase in temperature (Table 2).

**Table 2:** Effect of different orange peel concentration on Emulsification activity and biosurfactant production by *B. licheniformis*

Orange peel concentration (%)	Emulsification index (%)*	Biosurfactant yield (g/L)*	Temperature (°C)	Emulsification index (%)*	Biosurfactant yield (g/L)*
1	60.38 ± 1.615	0.78 ± 0.052	25	57.84 ± 2.408	0.621 ± 0.038
2	71.08 ± 1.076	1.232 ± 0.072	30	70.75 ± 1.051	1.283 ± 0.020
3	71.97 ± 1.279	1.387 ± 0.051	35	69.55 ± 0.852	1.193 ± 0.015
4	75.17 ± 0.390	1.796 ± 0.011	40	60.06 ± 1.014	0.862 ± 0.017
5	73.21 ± 1.098	1.585 ± 0.099	45	19.58 ± 0.854	0.146 ± 0.015

* Mean ± SD, no = 3

### Characterization of biosurfactant from *B. licheniformis*

#### TLC and high pressure liquid chromatography (HPLC) analysis

**Fig. 4:**
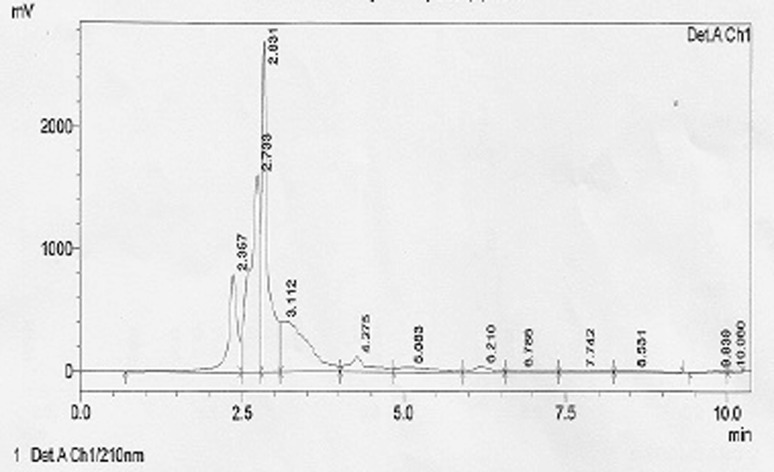
HPLC chromatogram of the TLC fraction from *B. licheniformis*

The crude biosurfactant obtained after solvent extraction from the culture supernatant of *B. licheniformis* was further purified by different chromatographic techniques. Crude extract of the biosurfactant was recovered from the culture supernatant of bacterial isolate by ethyl acetate extraction process. After acid precipitation crude biosurfactant was examined and separated using thin layer chromatography (TLC). Single spot was observed at an Rf value 0.56. The TLC fraction was further successfully purified by HPLC column at different elution times and is presented in Fig. 4. In HPLC analysis, three peaks were observed at retention times of 2.3, 2.5 and 2.8. The purified fraction was analyzed for further characterization of biosurfactant from *B. licheniformis*.

### Biochemical composition of biosurfactant produced by *B. licheniformis*

The biosurfactant compound showed negative result towards the bromine water reaction indicating that the fatty acid chain was saturated. In the same way it showed negative result to ninhydrin reaction indicating that the peptide has a blocked N-terminal. Biuret reaction was positive indicates the presence of polypeptides. The above results indicate that the biosurfactant has a lipopeptide in nature. Similar types of findings were reported in *B. subtilis* producing lipopeptide (Ishigami 1997; Dehghan-Noudeh et al. 2005).

### FTIR analysis of biosurfactant

**Fig. 5:**
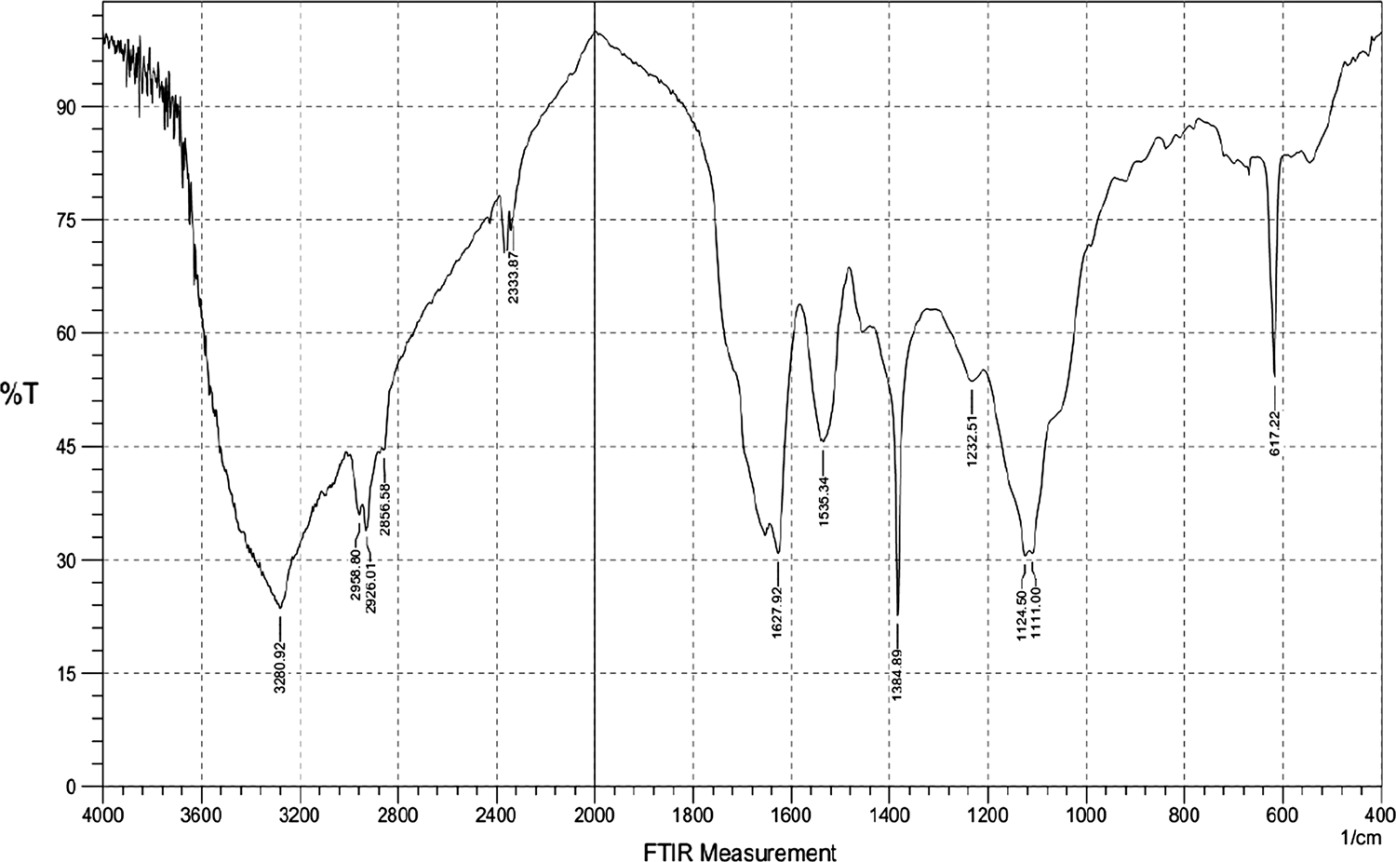
FTIR spectrum analysis of biosurfactant produced by *B. licheniformis*

The IR spectrum analysis of biosurfactant from *B. licheniformis* showed strong bands, indicating the presence of a peptide component at 3280.92 cm^−1^ resulting from the N–H stretching mode, at 1627.92 cm^−1^ resulting from the stretching mode of the CO–N bond, and at 1535.34 cm^−1^ resulting from the deformation mode of the N–H bond combined with the C–N stretching mode. The bands at 2958–2856 and 1384 cm^−1^ resulting from the C–H stretching mode suggest the presence of an aliphatic chain. These results were strong evidence that lipopeptide contains aliphatic and peptide likes moieties. The band at 1735 cm^−1^ was due to lactone carbonyl absorption (Fig. 5). These patterns were similar to those of lipopeptide compound, i.e., surfactin and lichenysin (Yakimov et al. 1995; Lin et al. 1998; Joshi et al. 2008).

### NMR spectroscopy analysis of biosurfactant from a culture of *B. licheniformis*

**Fig. 6:**
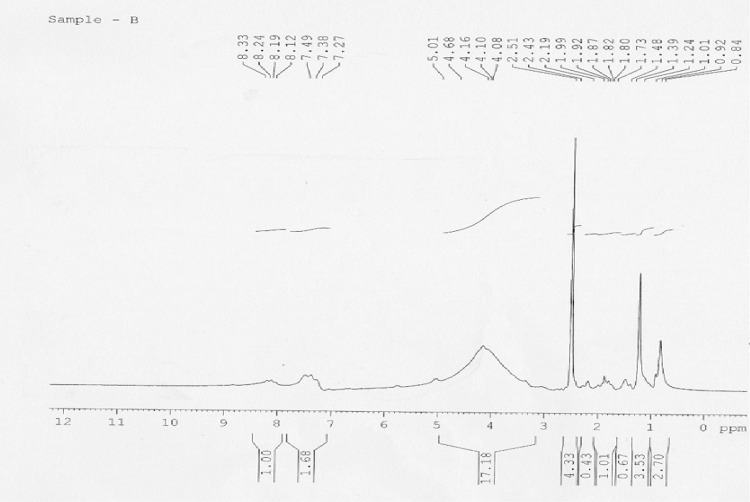
Chemical shift assignments of biosurfactant in ^1^H NMR spectra produced by *B. licheniformis*

The ^1^H-NMR of the extracted biosurfactant from *B. licheniformis* is presented in Fig. 6. A doublet signal at 0.8–0.9 ppm corresponding to six hydrogen atoms suggests the presence of a terminal isopropyl group. The assignments of other signals are given in Fig. 6. The spectra showed two main regions corresponding to resonance of α-carbon protons (3.5–5.5 ppm) and side-chain protons (0.25–3.0 ppm) (Peypoux et al. 1991; Chen and Juang 2008).

The chemical shift at 4.1 ppm observed in the extracted biosurfactant from *B. licheniformis* is similar with that observed in ^1^H NMR spectra of lipopeptide monoesters previously reported in literature (Tang et al. 2007; Kowall et al. 1998; Liu et al. 2009), thus suggesting the presence of one O-CH_3_ group. The presence of the methyl esters in the structure of surfactins has been related with an increase of its hydrophobicity and, consequently an increment of their surfactant powers, antifungal and hemolytic activities (Kowall et al. 1998). On the other hand, a chemical shift at 5.01 ppm was observed which corresponds to the H-3 (terminal proton) of the fatty acid chain, which has also been previously observed by other authors (Kowall et al. 1998; Liu et al. 2009). All the data suggests that the fatty acid residue from lipopeptide.

### Biodegradation studies of naphthalene and crude oil

**Fig. 7:**
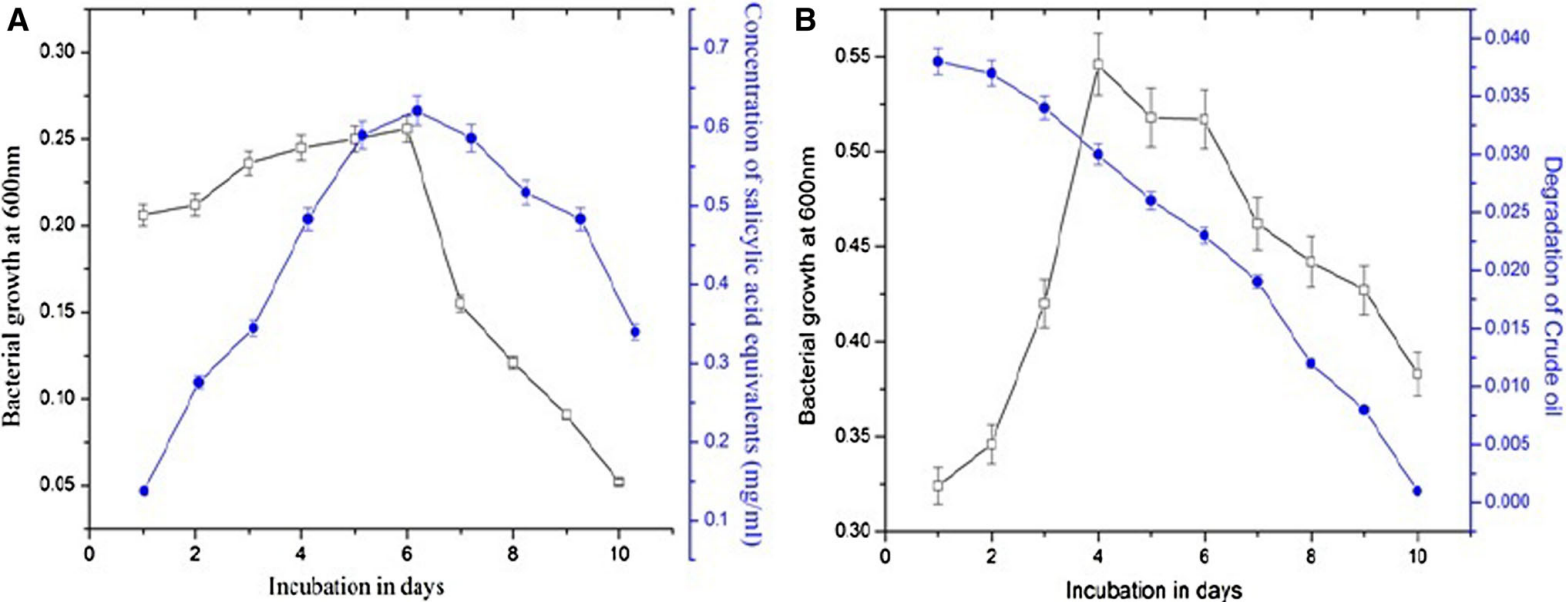
Biodegradation of naphthalene (**a**) and crude oil (**b**) by *B. licheniformis*

In this study, the potent bacterial isolate *B. licheniformis* were used for the naphthalene degradation studies. The bacterial degradation of naphthalene was quantified indirectly by the determination of naphthalene polar intermediates, i.e., salicylic acid. Accumulation of the major metabolite salicylic acid was started slowly by day 1 onwards and it reached maximum at 6th day of incubation for *B. licheniformis* and later it was decreased (Fig. 7a). In the earlier studies, the production of biosurfactants by microorganisms which can reduce the surface tension of the broth and subsequently increases the bioavailability of the naphthalene to the bacteria. In general the mechanism of aerobic degradation of Naphthalene involves the incorporation of molecular oxygen into one of the aromatic rings by Naphthalene dioxygenase, leading to the formation of cis-1,2-Naphthalene dihydrodiol. The later undergoes a number of further degradative steps and finally gets metabolized to carbon dioxide through salicylic acid (Seo et al. 2009). Naphthalene biodegradation is the best studied PAHs because it has simple structure and most soluble in nature and naphthalene-degrading microorganisms are relatively easy to isolate. Recently Dasari et al. (2014), reported that *P. aeruginosa* degrade the naphthalene in presence of biosurfactant at 6th day of incubation.

In the crude oil degradation studies, it showed high degradation levels (Fig. 7b). Simultaneously, we also checked the growth of the organism. In the growth study, *B. licheniformis* showed maximum growth at 4th day of incubation and later onwards the growth was declined. Generally microorganisms can convert the crude oil chemical compounds into for their energy, cell mass and biological products (Zhang et al. 2005). Based on these biodegradation studies *B. licheniformis* adds to its value as an ecofriendly and biodegradable product. Mohammad et al. (1985), stated that the biosurfactant produced from *B. licheniformis* JF-2 has ability to enhanced oil recovery process.

The maximum percentage of bacterial adherence of 32.57 % was reported by *B. licheniformis* with crude oil. The BATH assay was confirmed by visualization of cells adhered to crude oil and it was confirmed by the affinity of cells towards crude oil facilitated by producing biosurfactant (Thavasi et al. 2008). The hydrocarbon degraders normally produce surfactants which adhere effectively to hydrophobic substrate (Nishanthi et al. 2010). Pruthi and Cameotra (1997), showed that the ability of bacteria to adhere to hydrocarbons is a characteristic feature of biosurfactant producing microbes. According to Thavasi et al. (2011), the bacterial isolates with high hydrophobicity are likely to be more efficient degraders. Franzetti et al. (2009) stated that the cell hydrophobicity is also an indication of biosurfactant production.

## Conclusion

A Gram-positive bacterium was isolated from mangrove sediment soil and it was identified as *B. licheniformis* with an accession number KC710973. The orange peel substrate was utilized by *B. licheniformis* as a very effective carbon source and biosurfactant production. From HPLC, FTIR and NMR studies the biosurfactant revealed that the compound was lipopeptide. From the biodegradation studies, it concluded that the biosurfactant produced by *B. licheniformis* further can add to its value as an ecofriendly and biodegradable product.

## References

[CR1] Abioye OP, Agamuthu P, Abdul Aziz AR (2012). Biodegradation of used motor oil in soil using organic waste amendments. Biotechnol Res Int.

[CR2] Abu-Ruwaida AS, Banat IM, Haditirto S, Salem A, Kadri M (1991). Isolation of biosurfactant-producing bacteria product characterization and evaluation. Act Biotechnologica.

[CR3] Altschul SF, Thomas LM, Alejandro AS, Zhang JH, Zhang Z, Webb M (1997). Gapped BLAST and PSI-BLAST: a new generation of protein database search programs. Nucleic Acids Res.

[CR4] Arutchelvi J, Doble M (2010). Characterization of glycolipid biosurfactant from *Pseudomonas aeruginosa* CPCL isolated from petroleum-contaminated soil. Lett Appl Microbiol.

[CR5] Banat IM (1993). The isolation of a thermophilic biosurfactant producing *Bacillus* sp.. Biotechnol Lett.

[CR6] Bernard D, Pascaline H, Jeremie JJ (1996). Distribution and origin of hydrocarbons in sediments from lagoons with fringing mangrove communities. Mar Pollut Bull.

[CR7] Blodgett WC (2001). Water-soluble mutagen production during the bio-remediation of oil-contaminated soil. Fla Sci.

[CR8] Boonchan S, Britz ML, Stanley GA (2000). Degradation and mineralization of high-molecular-weight polycyclic aromatic hydrocarbons by defined fungal-bacterial cocultures. Appl Environ Microbiol.

[CR9] Box JD (1983). Investigation of the Folin-Ciocalteu phenol reagent for the determination of polyphenolic substances in natural waters. Water Res.

[CR10] Carrillo P, Mardaraz C, Pitta-Alvarez S (1996). Isolation and selection of biosurfactant-producing bacteria. World J Microbiol Biotechnol.

[CR11] Cerniglia CE (1992). Biodegradation of polycyclic aromatic hydrocarbons. Biodegradation.

[CR12] Chen HL, Juang RS (2008). Recovery and separation of surfactin from pretreated fermentation broths by physical and chemical extraction. Biochem Eng J.

[CR13] Dasari S, Venkata Subbaiah KC, Rajendra W, Lokanatha V (2014). Biosurfactant-mediated biodegradation of polycyclic aromatic hydrocarbons—naphthalene. Bioremed J.

[CR14] Dehghan-Noudeh G, Housaindokht M, Bazzaz BSF (2005). Isolation, characterization, and investigation of surface and hemolytic activities of a lipopeptide biosurfactant produced by *Bacillus subtilis* ATCC 6633. J Microbiol.

[CR15] Feignier C, Besson F, Michel G (1995). Studies on lipopeptide biosynthesis by *Bacillus subtilis*: isolation and characterization of iturin, surfactin mutants. FEMS Microbiol Lett.

[CR16] Franzetti A, Caredda P, Colla PL, Pintus M, Tamburini E, Papacchini M, Bestetti G (2009). Cultural factors affecting biosurfactant production by *Gordonia* sp. BS29. Int Biodeterior Biodegrad.

[CR17] George S, Jayachandran K (2009). Analysis of rhamnolipid biosurfactants produced through submerged fermentation using orange fruit peelings as sole carbon source. Appl Biochem Biotechnol.

[CR18] Hagwell LS, Delfino LM, Rao JJ (1992). Partitioning of polycyclic aromatic hydrocarbons from diesel fuel into water. Environ Sci Technol.

[CR19] Ishigami Y, Esmi K (1997). Characterization of biosurfactants. Structure performance relationships in surfac-tants.

[CR20] Johnson M, Boese-Marrazzo D (1980). Production and properties of heat stable extracellular hemolysin from *Pseudomonas aeruginosa*. Infect Immun.

[CR21] Joshi S, Bharucha C, Sujata J, Yadav Sanjay, Anuradha N, Desai AJ (2008). Biosurfactant production using molasses and whey under thermophilic conditions. Bioresour Technol.

[CR22] Kiran GS, Hema TA, Gandhimathi R, Selvin J, Manilal A, Sujith S, Natarajaseenivasan K (2009). Optimization and production of a biosurfactant from the sponge-associated marine fungus *Aspergillus ustus* MSF3. Colloids Surf B Biointerf.

[CR23] Kokare CR, Kadam SS, Mahadik KR, Chopade BA (2007). Studies on bioemulsifier production from marine *Streptomyces* sp.S1. Ind J Biotechnol.

[CR24] Kowall M, Vater J, Kluge B, Stein T, Franke P, Ziessow D (1998). Separation and characterization of surfactin isoforms produced by *Bacillus subtilis* OKB 105. J Colloid Interface Sci.

[CR25] Lin SC, Lin Kuo-Ging, Lo Chih-Chen, Lin Yu-Ming (1998). Enhanced biosurfactant production by a *Bacillus licheniformis* mutant. Enz Microbial Technol.

[CR26] Liu XY, Yang SZ, Mu BZ (2009). Production and characterization of a C-15-surfactin-*O*-methyl ester by a lipopeptide producing strain *Bacillus subtilis* HSO121. Proc Biochem.

[CR27] Lowry OH, Rosebrough NJ, Farr AL, Randall RJ (1951). Protein measurement with the Folin–Phenol reagents. J Biol Chem.

[CR28] Makkar RS, Cameotra SS (2002). An update on the use of unconventional substrates for biosurfactant production and their new applications. Appl Microbiol Biotechnol.

[CR29] Mihelcic JR, Lueking DR, Mitzell RJ, Stapleton JM (1993). Bioavailability of sorbed- and separate-phase chemicals. Biodegradation.

[CR30] Mohammad J, Jenneman GE, McInerney MJ, Knapp RM (1985). Anaerobic production of a biosurfactant by *Bacillus licheniformis* JF-2. Appl Environ Microbiol.

[CR31] Monteiro SA, Sassaki GL, de Souza LM, Meira JA, de Araujo JM, Mitchell DA, Ramos LP, Krieger N (2007). Molecular and structural characterization of the biosurfactant produced by Pseudomonas aeruginosa DAUPE 614. Chem Physics Lipids.

[CR32] Moran A, Alejandra M, Martinez F (2002). Quantification of surfactin in culture supernatant by hemolytic activity. Biotechnol Lett.

[CR33] Nishanthi R, Kumaran S, Palani P, Chellaram C, Prem Anand T, Kannan V (2010). Screening of biosurfactants from hydrocarbon degrading bacteria. J Ecobiotechnol.

[CR34] Perfumo A, Banat IM, Canganella F, Marchant R (2006). Rhamnolipid production by a novel thermotolerant hydrocarbon-degrading *Pseudomonas aeruginosa* AP02-1. Appl Microbiol Biotechnol.

[CR35] Peypoux F, Bonmatin JM, Labbé H, Das BC, Ptak M, Michel G (1991). Isolation and characterization of a new variant of surfactin, the [Val7]surfactin. Eur J Biochem.

[CR36] Pruthi V, Cameotra S (1997). Rapid identification of biosurfactant-producing bacterial strains using a cell surface hydrophobicity technique. Biotechnol Tech.

[CR37] Rahman KSM, Banat IM, Thahira J, Thayumanavan T, Lakshmanaperumalsamy P (2002). Bioremediation of gasoline contaminated soil by a bacterial consortium amended with poultry litter, coir pith and rhamnolipid biosurfactant. Biores Technol.

[CR38] Reda AB, El-Nagar AY (2009). Safe control methods of petroleum crude oil pollution in the mangrove forests of the Egyptian red sea coast. J Appl Sci Res.

[CR39] Rosenberg M, Gutnick DL, Rosenberg E (1980). Adherence of bacteria to hydrocarbons: a simple method for measuring cell-surface hydrophobicity. FEMS Microbiol Lett.

[CR40] Saimmai A, Rukadee O, Onlamool T, Sobhon V, Maneerat S (2012). Isolation and functional characterization of a biosurfactant produced by a new and promising strain of *Oleomonas sagaranensis* AT18. World J Microbiol Biotechnol.

[CR41] Saimmai A, Sobhon V, Maneerat S (2012). Production of biosurfactant from a new and promising strain of *Leucobacter komagatae* 183. Ann Microbiol.

[CR42] Saimmai A, Tani A, Sobhon V, Maneerat S (2012). Mangrove sediment, a new source of potential biosurfactant producing bacteria. Ann Microbiol.

[CR43] Satpute SK, Banpurkar AG, Dhakephalkar PK, Banat IM, Chopade BA (2010). Methods for investigating biosurfactants and bioemulsifiers: a review. Crit Rev Biotechnol.

[CR44] Seo J-S, Keum Y-S, Li QX (2009). Bacterial degradation of aromatic compounds. Int J Environ Res Public Health.

[CR45] Singh A, Van Hamme JD, Ward PO (2007). Surfactants in microbiology and biotechnology. Part 2. Application aspects. Biotechnol Adv.

[CR46] Suwansukho P, Rukachisirikul V, Kawai F, H-Kittikun A (2008). Production and applications of biosurfactant from *Bacillus subtilis* MUV4. Songklanakarin J Sci Technol.

[CR47] Tabatabaee A, Assadi MM, Noohi AA, Sajadian VA (2005). Isolation of biosurfactant producing bacteria from oil reservoirs. Iran J Environ Health Sci Eng.

[CR48] Tamura K, Dudley J, Nei M, Kumar S (2007). MEGA4: molecular evolutionary genetics analysis (MEGA) software version 4.0. Mol Biol Evol.

[CR49] Tang JS, Gao H, Hong K, Yu Y, Jiang MM, Lin HP (2007). Complete assignments of ^1^H and ^13^C NMR spectral data of nine surfactin isomers. Magn Reson Chem.

[CR50] Thavasi R, Jayalakshmi S, Balasubramanian T, Banat IM (2008). Production and characterization of a glycolipid biosurfactant from *Bacillus megaterium* using economically cheaper sources. World J Microbiol Biotechnol.

[CR51] Thavasi R, Jayalakshmi S, Banat IM (2011). Application of biosurfactant produced from peanut oil cake by *Lactobacillus delbrueckii* in biodegradation of crude oil. Bioresour Technol.

[CR52] Yakimov MM, Timmis KN, Wray V, Fredrickson HL (1995). Characterization of a new lipopeptide surfactant produced by thermotolerant and halotolerant subsurface *Bacillus licheniformis* BAS50. Appl Environ Microbiol.

[CR53] Youssef NH, Duncan KE, Nagle DP, Savage KN, Knapp RM, McInerney MJ (2004). Comparison of methods to detect biosurfactant production by diverse microorganism. J Microbiol Methods.

[CR54] Zhang G, Wu Y, Qian X, Merg Qin (2005). Biodegradation of crude oil by *Pseudomonas aeruginosa* in the presence of rhamnolipids. J Zhejiang Univ Sci B.

